# A manual corpus of annotated main findings of clinical case reports

**DOI:** 10.1093/database/bay143

**Published:** 2019-01-17

**Authors:** Neil R Smalheiser, Mengqi Luo, Sidharth Addepalli, Xiaokai Cui

**Affiliations:** 1 Department of Psychiatry and Psychiatric Institute, University of Illinois College of Medicine, Chicago, IL, USA; 2 School of Information Management, Wuhan University, Wuhan, Hubei, China; 3 School of Information Sciences, University of Illinois at Urbana-Champaign, Champaign, IL, USA

## Abstract

Clinical case reports are the `eyewitness reports’ of medicine and provide a valuable, unique, albeit noisy and underutilized type of evidence. Generally a case report has a single main finding that represents the reason for writing up the report in the first place. In the present study, we present the results of manual annotation carried out by two individuals on 500 randomly sampled case reports. This corpus contains main finding sentences extracted from title, abstract and full-text of the same article that can be regarded as semantically related and are often paraphrases. The final reconciled corpus of 416 articles comprises an open resource for further study. This is the first step in establishing text mining models and tools that can identify main finding sentences in an automated fashion, and in measuring quantitatively how similar any two main findings are. We envision that case reports in PubMed may be automatically indexed by main finding, so that users can carry out information queries for specific main findings (rather than general topics)—and given one case report, a user can retrieve those having the most similar main findings. The metric of main finding similarity may also potentially be relevant to the modeling of paraphrasing, summarization and entailment within the biomedical literature.

## Introduction

Clinical case reports are the `eyewitness reports’ of medicine, in which novel or interesting observations are made of one or a few patients. The major biomedical search engine, PubMed, indexes almost two million articles as having the Publication Type `case reports’, ~7% of all biomedical articles.

Case reports rank at the bottom in the hierarchy of evidence-based medicine, far below randomized controlled trials (1), and they are generally not included in the assessment of clinical evidence carried out by systematic reviews and meta-analyses. However, case reports provide a valuable, unique, albeit noisy and underutilized type of evidence (2, 3). In particular, given one case report, it would be desirable to retrieve other case reports that have reported similar findings (not just discussed similar topics). There is considerable value in identifying findings that have been independently published in multiple case reports, since that would alert readers to evidence that has particularly high reliability and potential impact (2). In turn, this might encourage wider judicious use of case reports in evidence-based medicine and other tasks such as surveillance of drug side-effects.

Manual annotation of clinical case reports has been pursued in previous studies that have extracted potential adverse drug event relations (4) and biomedical concepts (5). Corpora consisting of clinical case reports have been employed for training machine reading comprehension (6) and automated recognition of rare disease phenotypes (7). We hypothesize that case reports should be an ideal test bed for annotating main findings and for establishing text mining models and tools that can identify sentences that state main findings in an automated fashion. This is because generally a case report has a single main finding **that represents the reason for writing up and publishing the report in the first place**. As documented below, the title of the case report usually directly states the main finding, and this is restated one or more times in the abstract (if present) and in the full-text. Main findings tend to occur in particular locations, and are often found in sentences that begin `We report….’ or `In conclusion,...’. Thus, case reports should be a relatively easy type of article for identifying and mining main findings, in contrast to clinical trial articles or laboratory studies, which tend to be far more complex.

In the present study, we present the results of manual annotation carried out by two individuals on 500 randomly sampled case reports, which comprises an open resource for further study. This corpus contains paired sentences extracted from title, abstract and full-text of the same article that can be regarded as semantically related, and often paraphrases. Having such a corpus is the first step in creating models and tools that can identify main findings in an automated fashion. This corpus may also be of interest to the general issue in computational linguistics of measuring semantic similarity of sentences and other short texts (8, 9), similar to relating citances to citations (10) or studying how the truth of certain sentences can be inferred from other sentences (11, 12).

## Methods

### General guidelines.

**We define a sentence (or title) as stating a main finding if it expresses the finding that motivated the authors to write up the case report for publication.** Rosenthal (13) lists nine reasons for publishing a clinical case report:

- First report of a new entity
- Additional examples that establish an entity from an isolated observation
- A new diagnostic test (either imaging or histological)
- Clinical behavior contrary to expectations based upon what we think we know
- Novel treatment with outcome
- A report of a well-described but rare disease
- An example of rare (<5) but not necessarily unexpected behavior in any condition
- A report of an uncommon disease (10–15 cases already reported)
- A remarkably well-documented example of educational value.

Thus, a case report might be written up because it describes a new syndrome, an unusual clinical course or simply for its teaching value. The main finding describes that syndrome or patient presentation. For example, the title of one case report is `Shiitake Dermatitis After Consumption of Homemade Soup’ (PMID 29901501). This, in a nutshell, is its main finding.

It is important to distinguish main findings from many other types of findings made in biomedical articles. For example, what we call main findings is not the same as sentences that assert knowledge claims (14–16), sentences that are descriptions of clinical outcomes (17), sentences that summarize the article as a whole, or lists of topics, concept, or keywords that are discussed in the article. While this article was under review, Shardlow *et al.* (18) identified sentences that present New Knowledge (an author’s findings). This is similar to our idea insofar as the main finding of a case report represents a particular context for presenting New Knowledge. However, note that the sentence that states the main finding is generally NOT the same as the sentence that states the `take home message’—the latter provides context for the main finding, elaborates on it, asserts its importance or points out implications for clinicians.

We hypothesized that a clinical case report will almost always state its main finding explicitly, and almost always, there will be a single main finding per article. (There generally is, however, more than one statement of this main finding within the same article.) It is expected that the main finding will be expressed in a single sentence, or uncommonly, across two adjacent sentences that express one thought. In the latter case, both sentences should be annotated as the main finding.

### Annotation overview

As of May 2018, there were 1469 articles indexed as Case Reports [Publication Type] in PubMed, written in English or having English abstract, published between 1987–2017, which had title, abstract and full-text freely available in PubMed Central. We randomly chose 500 of these articles to be manually annotated by two students, SA and XC. SA is a layperson with no specialized training in medicine, whereas XC completed a master’s program in veterinary science. Both are non-native speakers but fluent in English. After several training sessions on case reports not included here, the annotators marked up the articles independently. Each week (after annotating roughly 70–100 articles), the team looked over the results together and corrected any errors that reflected systematic or training issues, to ensure that criteria were being applied fairly and consistently.

A main finding is often stated and restated in different ways in multiple places within an article. We want to capture the main finding in the title (if present), in the abstract (if present), and in the full-text (if present). Within each of these locations, we chose the `best’ statement of the main finding. If more than one sentence seemed equally appropriate, we chose one as the `best’ and a second one as the `alternate choice’. Sentences were cut-and-pasted by annotators from online text to an Excel spreadsheet, making sure that words were not separated by line returns, and excluded extraneous text such as numbers of cited references or pagination.

### Annotation of main findings in titles

For each article, its PubMed Central Identifier (PMC ID) was recorded and its title was entered verbatim. Each title was scored by each annotator as being

- a direct statement of the main finding (Y),
- alluding to the main finding but not a direct statement of it (A),
- NOT a statement of the main finding (N) or
- Uncertain (U).

Example of a title that states a main finding: `Shiitake mushroom-induced flagellate dermatitis.’

Example of a title that alludes to the main finding without actually stating it: `A 5-year-old girl with decreased vision in the left eye.’ We say that this ALLUDES to the main finding because it is unlikely that observing a 5-year-old girl with decreased vision in the left eye would be sufficient motivation, in itself, to write up a case report. Some additional finding(s), not stated in the title, must have been involved. This type of title is particularly common in case reports that are intended to serve educational purposes, as opposed to presenting new findings of scientific interest. Another example in which the title alludes to the main finding is `Response to Treatment X’. We say this ALLUDES because the nature of the response is not explicitly stated in the title (positive? negative? adverse?). Admittedly, the distinction between stating and alluding to main findings can sometimes be subtle or subjective.

Example of a title that does NOT state a main finding: `A culinary quandary?’

Example of a title that was marked as Uncertain: `An autopsy report on multiple system atrophy diagnosed immunohistochemically despite severe ischaemic damage: a new approach for investigation of medical practice associated deaths in Japan.’

Note that some titles are compound, e.g. `XYZ: A Case Report’. In such a case, supposing only XYZ actually states the main finding, we would still annotate the entire title since the presence of `a case report’ may be a useful feature for modeling the features that discriminate titles that do, vs. that do not, state main findings.

We also asked the annotators to mark whether the case reports were `typical’ or not. An example of a non-typical case report is `Cure or control: complying with biomedical regime of diabetes in Cameroon.’ Another is `Case studies in cholera: lessons in medical history and science.’ Such articles do not deal with clinical or scientific issues, but rather deal with e.g. policy, history or law.

### Annotation of main findings in abstracts

Only about half of case report articles in PubMed overall have abstracts that are marked as such, though the first paragraph of full-text often serves a similar purpose in articles that lack defined abstracts. Our corpus was restricted to articles that contained abstracts.

For each article, each annotator indicated whether

- the abstract contains a statement of the main finding or not (or uncertain)
- the abstract is structured or unstructured, and (for a structured abstract) the section of the abstract that contains the main finding sentence.

If a statement of the main finding is present, the entire sentence containing the statement is annotated as the main finding.

If two adjacent sentences expressed the main finding as a single thought, both sentences were annotated as a single main finding. For example, in article PMC4518556, the title expresses the main finding:

`Abatacept: a new treatment option for refractory adult autoimmune enteropathy’.

However, the abstract expresses the same thought over two adjacent sentences:

`We report a case of a 49-year-old woman who presented with refractory diarrhea, diagnosed as AIE. After failing multiple conventional therapies, she demonstrated clinical and histologic response to abatacept, a selective modulator of T-cell activation.’

If more than one sentence represents a complete statement of the main finding, then one was annotated as the `best’ example and the other(s) were annotated as alternate choices.

We expected that the most likely places to see the main finding within an unstructured abstract are the first and last sentence. Statements of the main finding often begin with `In conclusion…’, `These findings…’, `We report…’, `We present…’ or `Therefore,…’. The most likely location is within the Conclusions section of structured abstracts, or near the end of unstructured abstracts.

For articles in which the title states the main finding, we expect that the main finding expressed in the title and expressed in the abstract will have some obvious relationship to each other. Namely, the sentence found within the abstract should restate the title, perhaps in more detail. Articles in which the title and abstract appear to state entirely different main findings should be flagged for greater scrutiny.

### Annotation of main findings within the body (full-text) of clinical case reports.

For each article, each annotator examined the Introduction section (i.e. the first section or the first paragraph after the abstract) and indicated whether the section contains a statement of the main finding or not (or uncertain). If present, the sentence(s) stating the main finding were annotated. It is expected that the main finding as stated in full-text will be clearly related to the statements of main finding that may be present in title and in abstract of the same article. Each annotator also extracted main findings from the Discussion and Conclusions sections of full-text—or if these are not labeled as distinct sections, then the last paragraph of the article.

**Table 1:** Examples of titles and abstract main finding sentences

**PMC ID**	**Title**	**Abstract main finding**
PMC3015703	Bile causing an acute scrotum immediately after laparoscopic cholecystectomy.	To the best of our knowledge this is the first report of bile causing an acute scrotum following laparoscopic surgery.
PMC4913196	Combined Open-Heart Coronary Artery Bypass Surgery and Subtotal Thyroidectomy in a 54-year-old patient: A Case Report.	The evidence of the case showed that combined CABG and thyroidectomy can be performed safely.
PMC2576163	Persistent left superior vena cava: a case report and review of literature.	Persistent left superior vena cava is rare but important congenital vascular anomaly.
PMC4279607	Preoperative assessment of the older surgical patient: honing in on geriatric syndromes.	Here, we describe our initial two cases and review the stress response to surgery and the impact of advanced age on this response as well as preoperative geriatric assessments, including frailty, nutrition, physical function, cognition, and mood state tests that may better predict postoperative outcomes in older adults.
PMC5237170	Computed tomographic findings and treatment of a bull with pituitary gland abscess.	This report describes the clinical, computed tomographic and postmortem findings in a Holstein-Friesian bull with a hypophyseal abscess.
PMC3536036	Patterns of response in patients with pretreated metastatic melanoma who received ipilimumab 3 mg/kg in a European expanded access program: five illustrative case reports.	Here, case reports from five patients treated within an expanded access program (EAP) with ipilimumab at its licensed dose of 3 mg/kg illustrate the efficacy of ipilimumab in an expanded access setting and the range of different tumor response patterns encountered.
PMC2729415	Successful medical management of status post-Roux-en-Y-gastric-bypass hyperinsulinemic hypoglycemia.	In this letter, we describe the first successful management of status post-gastric-bypass hyperinsulinemic hypoglycemia with diazoxide.
PMC4862050	Finding a new therapeutic approach for no-option Parkinsonisms: mesenchymal stromal cells for progressive supranuclear palsy.	We used MSC as a novel candidate therapeutic tool in a pilot phase-I study for patients affected by progressive supranuclear palsy (PSP), a rare, severe and no-option form of Parkinsonism.
PMC3026672	Expanding the clinical spectrum of 3-phosphoglycerate dehydrogenase deficiency.	Here, we report for the first time a very mild form of genetically confirmed 3-PGDH deficiency in two siblings with juvenile onset of absence seizures and mild developmental delay.
PMC4007146	Combined endoscopic surgery in the prone-split leg position for successful single-session removal of an encrusted ureteral stent: a case report.	This is the first report describing the management of an encrusted stent using combined endoscopic surgery in the prone split-leg position in a single session.

Shown are ten examples taken from Supplemental File 2 chosen at random.

## Results

Supplemental File 1 displays the annotations resulting from discussion and corrections between the two annotators. (Of the 500 articles initially annotated, one article was excluded because the PMC ID was incorrectly cross-listed in PubMed.) There were a total of 416 articles in which both annotators agreed upon: (i) the article was a typical case report, (ii) the title either directly expressed or alluded to the main finding, (iii) the abstract expressed a main finding and (iv) both agreed which sentence(s) expressed the main finding within the abstract. This `cleaned up’ corpus is presented in Supplemental File 2. The full-text was more difficult to annotate than the abstract, in part because there were often multiple expressions of the main finding in different places, and in part because discussions often mixed statements of main finding with take-home lessons in a complex manner. No attempt was made to reconcile differences in markup of sentences found in full text; rather, all choices of both annotators are presented side by side in the corpus.

Only 13 of 500 articles were marked as `not typical’ case reports by one or both annotators. In the vast majority (91.1%) of articles, both annotators agreed that the title expressed the main finding explicitly. Cohen’s kappa equals 0.882 = near perfect agreement on titles.

Working independently, the annotators agreed on the abstract main finding in 322/500 = 64.4% of cases. Cohen’s kappa before discussions = 0.593 that represents `moderate agreement’. No systematic change in overall agreement was observed from the beginning to the end of the annotation process, suggesting that disagreements were judgment calls rather than cases of inadequate training. After discussion between the two annotators, the agreement increased to 448/500 = 89.6% of cases. The Cohen’s kappa after discussion equals 0.881 which is `near perfect agreement’ on abstract main findings. The extent of initial agreement, and agreement after discussions, are indicators of the difficulty of the annotation task. Thus, extracting the main finding manually was a relatively easy task in most cases.

We validated our initial expectations that each abstract had only one main finding: only 12.7% of articles (53 out of 416) were marked by at least one annotator as having a second main finding sentence in the abstract, and both annotators chose the same second sentence in only 5 of those 53 articles (Supplemental File 2). As well, the abstract main finding was expressed within one sentence in the vast majority of articles—only in 2.9% of articles (12 out of 416) did both annotators mark two adjacent sentences as stating a single main finding.

Some examples of titles and corresponding abstract main finding sentences are shown in Table 1. Although the abstract main finding often does paraphrase the title, there is a great variation—often the abstract main finding provides more detailed information, and often introduces domain-specific or potentially ambiguous abbreviations such as MSC for mesenchymal stem cells (but could also be marrow stromal cells). Thus, there is limited overlap in word token usage between the title and its corresponding abstract main finding.

Interestingly, the statements of main finding extracted from the full-text are rarely verbatim repeats of the abstract main finding. For example, in article PMC3026672, the abstract main finding is

`Here, we report for the first time a very mild form of genetically confirmed 3-PGDH deficiency in two siblings with juvenile onset of absence seizures and mild developmental delay.’ (Table 1).

In contrast, both annotators marked the full-text Introduction main finding sentence as

`In this paper we present a family with a hitherto unreported very mild phenotype of 3-PGDH deficiency, expanding the clinical phenotype to that of juvenile onset of seizures with mild psychomotor retardation.’

Another example is article PMC3015703, whose abstract main finding is

`To the best of our knowledge this is the first report of bile causing an acute scrotum following laparoscopic surgery.’ (Table 1).

Both annotators marked the full-text Introduction main finding as

`To the best of our knowledge, this is the first case of an acute hemiscrotum presenting after laparoscopic biliary surgery.’

And both marked the full-text Discussion main finding sentence as

`We have described the presentation of an acute right hemiscrotum immediately following laparoscopic cholecystectomy.’

The variations in word usage, word order and detail are constrained and yet substantial and informative.

## Discussion

Our corpus was not sampled in an unbiased manner from the entire set of almost 2 million case reports indexed in PubMed. Rather, we sampled only from case reports having abstracts and whose full-text is freely available in XML format in PubMed Central. Although the range of journals may be narrower, we think it is likely that our sample is still representative of the way that case reports are generally written up, and in particular, how titles are crafted and how abstracts and full-text relate to the titles. Verspoor *et al*. (19) have found, reassuringly, that open access articles resemble non-open access articles in their linguistic characteristics.

Having the entire articles readily available in electronic form will greatly facilitate the use of this corpus for subsequent text mining and machine learning analyses. For example, the paired title/abstract/full-text sentences from the same articles are all positive examples of paraphrases. Supplemental File 3 contains negative examples paired to the abstract main finding sentences of the final corpus (Supplemental File 2), and a similar approach could choose negative examples for the annotated main finding sentences from the same section or same paragraph of the full-text. Having the article in electronic form will also facilitate extracting linguistic features of the main finding vs. negative sentences themselves, e.g. the number of words in the sentence, the verbs and verb tenses used, specific words and phrases employed, positive vs. negative sentiment, etc. We can also extract metadata features from the articles themselves, e.g. the Medical Subject Heading terms, as well as derived features that involve external resources, e.g. mapping terms in the sentences to concepts in the Unified Medical Language System or relations to knowledge graphs.

There is extensive literature on ways to index and extract information from PubMed records to facilitate faceted search and identify conceptually related articles (e.g. 20–23). We envision that case reports in PubMed may be automatically indexed by main finding, so that users can carry out information retrieval searching for specific main findings (rather than general topics). Because the title of a case report usually states the main finding in a concise fashion, one might think that the title alone could be used to index case reports by the main finding. However, this strategy would not generalize to other types of articles, for which the title does not generally convey the main finding. Equally important, it is likely that a composite/normalized main finding, synthesized by including both title and abstract main findings (and full-text main finding sentences when available), should result in a more informative and robust representation compared to use of the title alone.

Creating positive and negative training sets of paired titles and main finding sentences is not only the first step in automated identification of main findings within clinical case reports, but also the first step in creating a new metric of main finding similarity that describes how composite title/abstract/full-text main finding sentences are related to each other. Given one case report, we envision that a user could use the main finding similarity metric to retrieve the case reports having the most similar main findings (2). Note that main finding similarity may involve more than simple sentence similarity—sentences will probably need to undergo a semantic normalization or transformation process (12) (e.g. mapping to some ontologies), and this will require additional research. The metric of main finding similarity may also potentially be relevant to the more general modeling of paraphrasing, summarization, and entailment within the biomedical literature (24–26).

In the future, we hope to tackle the annotation of other types of biomedical articles, in particular, clinical trials and systematic reviews. Such articles are far more complex than case reports. For example, a clinical trial article may not explicitly state a hypothesis to be tested or the motivation for conducting the study. It may report multiple primary and secondary outcomes. There is often one sentence that summarizes the overall (positive or negative) outcome, and sentences that provide take-home lessons or implications, but none of these are as simple, direct, and straightforward as the main finding sentences of case reports. Thus, we will need to delineate a taxonomy of argumentation (27, 28) before annotating sentences in clinical trials and systematic reviews.

## Supplementary Material

Supplementary Data
